# Peak Vertical Ground Reaction Force during Two-Leg Landing: A Systematic Review and Mathematical Modeling

**DOI:** 10.1155/2014/126860

**Published:** 2014-08-26

**Authors:** Wenxin Niu, Tienan Feng, Chenghua Jiang, Ming Zhang

**Affiliations:** ^1^Tongji Hospital, Tongji University School of Medicine, Shanghai 200455, China; ^2^Interdisciplinary Division of Biomedical Engineering, The Hong Kong Polytechnic University, Hong Kong; ^3^Shanghai Key Laboratory of Orthopaedic Implants, Shanghai 200011, China; ^4^Department of Disaster and Emergency Medicine, Eastern Hospital, Tongji University School of Medicine, Shanghai 200120, China

## Abstract

*Objectives*. (1) To systematically review peak vertical ground reaction force (PvGRF) during two-leg drop landing from specific drop height (DH), (2) to construct a mathematical model describing correlations between PvGRF and DH, and (3) to analyze the effects of some factors on the pooled PvGRF regardless of DH. *Methods*. A computerized bibliographical search was conducted to extract PvGRF data on a single foot when participants landed with both feet from various DHs. An innovative mathematical model was constructed to analyze effects of gender, landing type, shoes, ankle stabilizers, surface stiffness and sample frequency on PvGRF based on the pooled data. *Results*. Pooled PvGRF and DH data of 26 articles showed that the square root function fits their relationship well. An experimental validation was also done on the regression equation for the medicum frequency. The PvGRF was not significantly affected by surface stiffness, but was significantly higher in men than women, the platform than suspended landing, the barefoot than shod condition, and ankle stabilizer than control condition, and higher than lower frequencies. *Conclusions*. The PvGRF and root DH showed a linear relationship. The mathematical modeling method with systematic review is helpful to analyze the influence factors during landing movement without considering DH.

## 1. Introduction

Landing movement has been thoroughly researched in sports biomechanics, because it is very important in gymnastics [1, 2], parachuting [3, 4], Parkour [5], volleyball [6, 7], basketball [8, 9], soccer [10], Australian football [11], and netball [12, 13]. In these studies, the landing risk or performance was valuated with various kinetic, kinematic, and electromyographic parameters, among which the ground reaction forces (GRF) is very important and fundamental [13, 14]. The GRF parameters are often compared between or among different participant groups or trial conditions to draw intuitional conclusions regarding biomechanical evaluation [15–21]. Because the vertical GRF (vGRF) is markedly larger compared to the anterior-posterior or medial-lateral component, its peak value (PvGRF) has been favored in most studies.

To realize different mechanical demands in laboratory, the drop height (DH) differed greatly in various studies. Hoffrén et al. [22] studied drop landing from a 10-cm DH, while Zhang et al. [23] measured GRF of landing from a 103-cm DH. The DH range was so large that PvGRF was also widely distributed. Even for the same DH of 60 cm as an example, the mean PvGRF ranged from 2.38 to 4.91 times body weight (BW) [1, 17, 24–34]. The wide range of PvGRF restricted the data comparison among various studies and the development of a consensus. It is necessary to comprehensively integrate and analyze the published PvGRF data during drop landing.

Though with a wide range, DH adopted in the controlled lab setting is still unavailable to reproduce most movements in the real world. To our knowledge, the maximum DH of drop landing performed in kinematic laboratory was 200 cm [35]. Also, fundamental backward rotating dismounts from beam have been measured with the mean peak centre of mass of 222 cm high [36]. However, the GRF data were not measured by the researchers. Considering safety issues, the DH higher than 100 cm was rarely adopted in laboratory. However, the practical height was higher than the testing DH in many sports, such as gymnastics [35] and parachuting [15]. The PvGRF is still unknown for a DH higher than the experimental limit [15]. This leads to invalid analyses for the practical condition. It is useful to predict PvGRF for any demanding landing based on current knowledge.

In 1942, De Haven [37] used the acceleration equation ($v=\sqrt{2gs}$) and an inversion of the equation for acceleration (*v* ^2^ = 2*gs*) to estimate the impact force in falls from heights. For the normal landing with autonomic posture regulation, Niu et al. [15] described the mean impact force determined by the initial landing velocity and buffer distance using an equation *G* = *V* ^2^/(2*Sg*), where *G*, *V*, *S*, and *g* represent mean impact force, initial velocity, buffer distance, and gravitational acceleration, respectively. Both methods are only used for estimating the mean force in the whole impact process but are unable to provide the peak value. Yeow et al. [38] determined regression relationships of DH with peak GRF, GRF slope, and impulse during landing. However, as there were only five participants, it is difficult to form a general conclusion. A large sample through various DHs is needed to predict the information when individuals land from a more practical DH.

A valid predicting method should be also helpful for comparing PvGRF between or among different groups or conditions. The landing biomechanics is often affected by the landing type [1, 23], instruction [14], shoe [39], ankle stabilizer [16], surface stiffness [2], and participant's age [40], sex [41], fatigue [42], and vision [43]. In many cases, researchers tried to find some evidences in PvGRF to evaluate certain influential factors, but many conflicting conclusions have been obtained in these studies. For example, some authors reported that women produced significantly higher PvGRF [6, 28, 29], while Blackburn and Padua [32] detected significantly higher PvGRF in men, and also some found no statistically significant differences in PvGRF between genders [8, 41, 42]. These contradictories may be related to different DHs in different studies. There was no effective method to compare the PvGRF data between different groups or landing conditions without considering DH. Based on abundant pooled data from systematic review, a mathematic modeling may provide some helpful clue to deal with this question.

Therefore, the purposes of this study were to (1) systematically review PvGRF data when participants landed from specific DHs with two legs; (2) construct a mathematical model describing the constitutive relationship between PvGRF and DH; and (3) analyze the effects of gender, landing type, shoes, ankle stabilizers, and surface stiffness on PvGRF regardless of DH based on the proposed models.

## 2. Material and Methods

### 2.1. Systematic Review

In April 2014, two independent reviewers (WN and TF) performed a computerized search for peer-reviewed journal articles published in English to identify studies reporting drop landing with both legs. Searches were performed using the following databases: PubMed, ScienceDirect, Ovid, and ISI Web of Knowledge. As the high performance force plate had seldom been used prior to 1980, the publication range was between 1980 and April 10, 2014. Keywords used included “landing” and “biomechanics” in combination. Furthermore, relevant articles were identified by cross-referencing the citation lists of the articles identified in the electronic search. In addition, authors in the field were contacted, and the authors also searched their own files.

Articles retrieved in the original search were exported into a single Endnote file (Thomson Reuters, Carlsbad, CA), and duplicate articles were removed. The title and abstract of each record were screened and irrelevant articles were excluded. Where insufficient information was available from the title and abstract, the full text was inspected. The remaining full-text articles were assessed for inclusion by one author (WN). Articles selected for exclusion were verified by another author (CJ), and any discrepancies were resolved through discussion involving all authors.

Studies that met the following criteria were included: (1) the study was conducted on humans; (2) the participant number was reported; (3) all participants were adults (12–60 yr), healthy, and with normal vision in the trials; (4) participants landed with both legs and stabilized themselves after contact, while the single-leg landing, countermovement-jump (CMJ) landing, or special type of landing (e.g., Parkour or parachuting roll fall) was excluded; (5) only the anticipated drop landing was included, while jump landing or unanticipated landing was excluded; (6) DH was reported; (7) PvGRF was provided with numerical presentation and was normalized to BW; (8) the impact force on one foot (left or right, dominant or nondominant) was independently measured with one force plate.

### 2.2. Mathematical Modeling

Using the principle of conservation of energy, gravitational potential energy equals the kinetic energy in the vertical direction. As described by de Haven [37], the impact velocity from DH in the ideal state can be calculated as follows:

(1) $$\begin{array}{c} v_{0}=\sqrt{2g\cdot \text{DH}}, \end{array}$$

where *v*_0_ is the impact velocity at initial contact and *g* is the gravitational acceleration. According to the law of conservation of momentum,

(2) $$\begin{array}{c} \overset{t}{\underset{0}{\int }}Fdt=\overset{0}{\underset{v_{0}}{\int }}mdv, \end{array}$$

where *F* is the vertical force on one leg and can be calculated as the difference of the measured vGRF and BW/2. At the time of *t* = 0, *v* = *v*_0_. In the impact process, *F* is a continuous function of the time (*t*). Based on the mean value theorem for integrals, there would exist a *F*′, such as *F*′Δ*t* = *mv*_0_. Then

(3) $$\begin{array}{c} \frac{F^{\prime }}{m}=v_{0};\\ m_{1}\text{PvGRF}=\sqrt{2g\cdot \text{DH}},\\ \text{PvGRF}=\frac{\sqrt{2g}}{m_{1}}\sqrt{\text{DH}}, \end{array}$$

where *m*_1_ is a modified parameter. Therefore, the relationship between PvGRF and DH was deduced as a square root form. At the end of the landing impact process, the subject gets his/her stability, and then *F* is about BW/2 rather than 0. Therefore, another parameter should be added into the square root equation. Then

(4) $$\begin{array}{c} \text{PvGRF}=a\sqrt{\text{DH}}+b, \end{array}$$

where $a\sim \sqrt{2g}/m_{1}$ and *b* ~ BW/2. The values of *a* and *b* can be calculated by the maximum likelihood method based on experimental data.

This mathematical model is proposed to illuminate the correlation between PvGRF and DH during a double-leg drop landing. Using the available data from pooled studies, the nonlinear regression can be transformed into linear regression in this model. R language team [44] and SPSS 17.0 (SPSS Inc., Chicago, IL, USA) were used to find the best fit and to statistically analyze the data. The *R* ^2^ value was adjusted based on the standard deviation and the sample count from each study, and it indicated a linear regression relationship between the independent and dependent variables, when it approached one. A *P* value less than 0.05 implied that the independent variable could be used to predict the dependent value. A smaller root mean squared error (RMSE) indicated a more accurate prediction.

To validate the mathematic model, we used the mean values of PvGRF measured by McNitt-Gray [45], in whose study, six gymnasts and six recreational athletes performed drop landings. Because the GRF data were measured on both feet together, they were not included in this systematic review. These data were used to validate our mathematic model for the following reasons: (1) they were not used to construct the model; (2) this study accords with the all other seven (1–7) criteria described above; and (3) one DH in this study was 128 cm and far beyond the maximum DH (103 cm) in our pooled data.

### 2.3. Influential Factors

If the gender was not explicitly reported or two genders were mixed in the same experiment, the data were excluded when the factor of gender was analyzed. Two types of drop landing with specific DHs have been customarily studied. The first is the suspended landing, meaning that participants are suspended above the force plate before dropping and are released to land autonomously. The second is the platform landing, meaning that participants initially stand on a platform and step or jump from it to land.

There were two conditions related to shoe condition: shod and barefoot. If this factor was not reported in the study, the data were excluded when shoe condition was analyzed. Two conditions were related to ankle protection: use of an ankle stabilizer (tape or brace) and a control without a stabilizer. Two conditions were classified according to the landing surface stiffness: hard and soft. If no mat or pad was used to cover the force plate, the surface was classified as hard. Three levels of sample frequency were considered as low (<1,000 Hz), medium (1,000–1,200 Hz), and high frequencies (>1,200 Hz).

When a participant dropped from a zero DH (DH = 0), then the formula would produce PvGRF = *b* (4). This critical condition actually represents static standing with two legs, which is seldom influenced by the other factors. Therefore, the variable *b* in the function is nearly unchangeable within various conditions. This means the variance of PvGRF at the same DH under different conditions could be explained by the variable *a*. Therefore, we used the modified PvGRF (mPvGRF), calculated by the below equation to determine the impact of other factors described above:

(5) $$\begin{array}{c} \text{mPvGRF}=\frac{\text{PvGRF}-\overset{-}{b}}{\sqrt{\text{DH}}}, \end{array}$$

where $\bar{b}$ is the weighted mean of *b*. Accordingly, data collected from different heights could be analyzed together. The mPvGRF was used to analyze the effects of various factors. The means and standard errors of PvGRF could be collected from all pooled papers. Based on the property of normal distribution, we could combine the datasets under the same condition and calculate the values of means and standard errors. In comparison between two conditions, the independent *t*-test was used to calculate the *P* value. In comparison among multiple conditions, analysis of variance (ANOVA) was used to calculate the *P* value.

## 3. Results

### 3.1. Systematic Review

**Figure 1:**
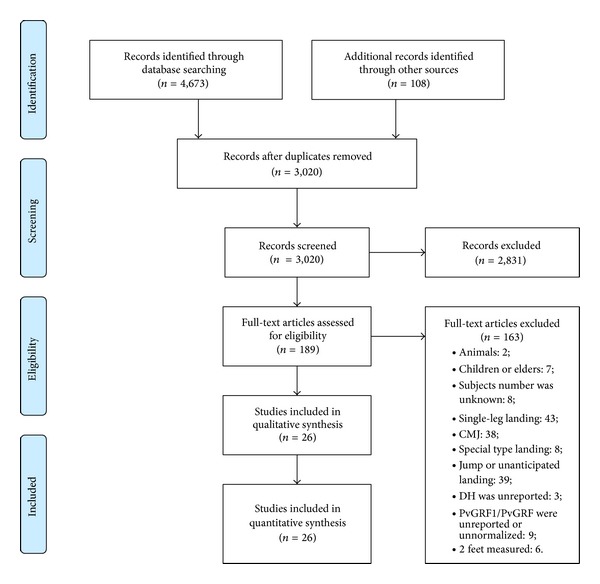
Identification of relevant publications (the PRISMA flow diagram).

The study selection process was described in Figure 1. The computerized literature search from all databases yielded 4,673 articles. After the removal of duplicates and irrelevant articles based on title and abstract screening, 189 articles remained, of which an additional 163 articles were removed on the basis of inclusion and exclusion criteria, leaving a final yield of 26 articles [1, 3, 15, 16, 18, 20, 22–34, 39, 42, 46–50]. The characteristics of all included articles were listed as Table 1.

Groups of male participants were measured in 16 articles [3, 15, 16, 23–26, 28, 29, 31–34, 42, 47, 48], while groups of female participants were measured in 11 articles [1, 3, 15, 16, 28–30, 32, 42, 46, 50]. In one article [27], the gender was not explicitly reported. In five articles [18, 20, 30, 39, 49], both genders were mixed in the same experimental group. In five articles [26, 29, 46, 49, 50], participants were suspended above the force plate before dropping. In the remaining 21 articles, the platform landing was studied.

In seven articles [1, 3, 15, 16, 18, 34, 50], the participants were shoeless. In thirteen articles [20, 24, 25, 27–29, 32, 39, 42, 47–49], the participants wore shoes. Shultz et al. [39] measured the same group of participants with and without shoes. In the other 7 articles [22, 25, 26, 30, 35, 48, 50], shoe conditions were not reported. The participants were measured without any ankle stabilizers in all 26 articles. In four articles [16, 20, 46, 49], the same groups of participants were also protected by ankle taping or bracing. Separate 1.6-cm rubber pads were used by Seegmiller and McCaw [1] to provide a nonslip, visually identical landing surface. In the other 25 articles, the landing surface was determined as hard, except that in one study [3], the hard surface and two types of soft surface were compared.

The sample frequencies were explicitly reported in all studies. Four studies were measured with sample frequencies <1,000 Hz [1, 24, 25, 46]. The frequencies ranged between 1,000 and 1,200 Hz in 20 articles [3, 15, 16, 18, 20, 23, 26–30, 32–34, 39, 42, 47–50]. Additionally, 2,000 Hz and 3,000 Hz were, respectively, reported by Hoffrén et al. [22] and Zhang et al. [31], who together provided eight groups of data with eight DH levels (15–90 cm).

### 3.2. Mathematical Modeling

**Figure 2:**
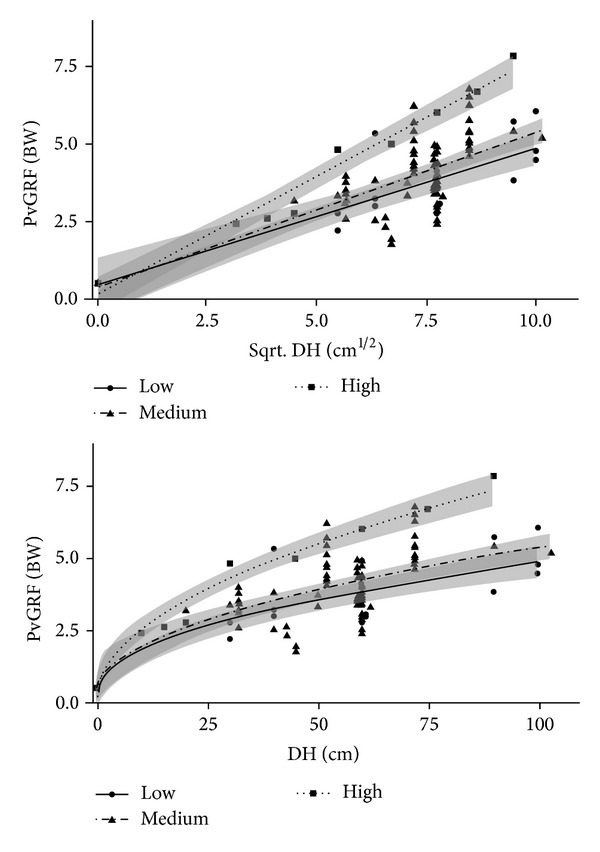
Regression between the sqrt. drop height (DH) and the peak vertical ground reaction force (PvGRF) classified by sample frequency (low frequency: $\text{PvGRF}=0.44\sqrt{\text{DH}}+0.45$, RMSE = 0.83, adjusted-*R* ^2^ = 0.73, *P* < 0.001; medium frequency: $\text{PvGRF}=0.49\sqrt{\text{DH}}+0.37$, RMSE = 0.94, adjusted-*R* ^2^ = 0.45, *P* < 0.001; high frequency: $\text{PvGRF}=0.75\sqrt{\text{DH}}+0.17$, RMSE = 0.46, adjusted-*R* ^2^ = 0.96, *P* < 0.001).

The regression results of the three groups divided by sampling frequency are shown in Figure 2. The RMSE values of the three regressions were 0.83, 0.37, and 0.46 for the low, medium, and high frequencies, respectively. The corresponding adjusted-*R* ^2^ values were 0.73, 0.94, and 0.46. The *P* value for each frequency was <0.001. The ANOVA found the statistical significance in the values of “*a*” among three regressions (*P* < 0.001). The difference of “*b*” among three regressions was not statistically significant (*P* = 0.35). The weighted mean of the three “*b*” values was 0.34.

**Figure 3:**
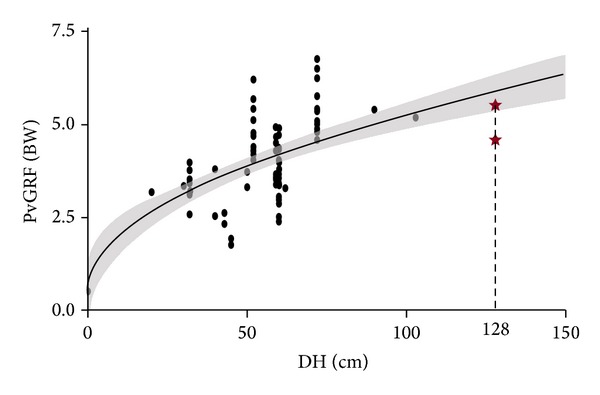
The validation of regression equation. Two star markers represent the measurement mean values of peak vertical ground reaction force (PvGRF) for the drop height of 128 cm. The shaded area represents the 95% confidence interval.

The data measured by McNitt-Gray [45] was used to validate the regression model. In his study, the sample frequency was 1,000 Hz, so the regression equation for the medium frequency ($\text{PvGRF}=0.49\sqrt{\text{DH}}+0.37$) was used to calculate the PvGRF. As shown in Figure 3, the calculated PvGRF mean was 5.9 BW for the 128 cm DH. McNitt-Gray [45] measured the PvGRF data on two feet, which were 11.0 and 9.1 BW for the gymnasts and recreational athletes, respectively. The calculated predictive value agreed with the half of experimental values well.

### 3.3. Influential Factors

**Figure 4:**
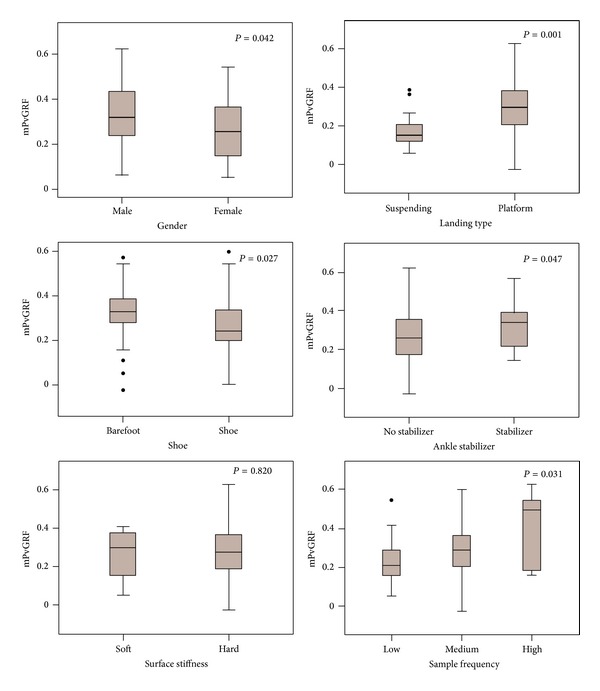
The effects of all involving factors on the modified peak vertical ground reaction force (mPvGRF).

The effects of all involved factors were listed in Figure 4. The PvGRF was not significantly affected by the surface stiffness but was significantly higher in men than women, the platform than suspended landing, the barefoot than shod condition, and ankle stabilizer than control condition and higher than lower sample frequencies.

## 4. Discussion

### 4.1. Systematic Review

We selected 26 articles to make this systematic review. As seen in Table 1 and Figure 2, the large ranges of PvGRF and DH were included in these pooled data. Though there are also many other similar articles, they do not completely meet the criteria. For example, some studies measured resultant GRF on double feet [5, 45, 51, 52]. According to Criterion 8, they were all excluded in our analysis. The standard of landing posture and trial process should be constructed for convenience of comparison among different studies.

Fifteen articles about single-leg landing were recently systematically reviewed to compare GRF parameters between patients with foot/ankle pathology and healthy controls [53]. They found that PvGRF yielded small significant pooled effect size of 0.38 but concluded the GRF parameters of time to stabilization further met the criteria for proven or candidate relevant parameters. Niu et al. [21] concluded that parameters of time to stabilization were not sensitive for two-leg landing. This study showed satisfying sensitivity when evaluating most influential factors during two-leg landing. However, more kinematic, kinetic, and electromyographic parameters should be systematically reviewed and evaluated to construct a full biomechanical view of this movement.

### 4.2. Mathematical Modeling

Yeow et al. [38] used a simple linear, exponential, and natural logarithmic function to fit the relationship of peak GRF and DH of the experimental data and finally found that they typically followed an exponential regression relationship of

(6) $$\begin{array}{c} y=ae^{bx}, \end{array}$$

where *y* = GRF and *x* = DH. The exponential function fit only considered the mathematical implications, but not the practical significance. For example, in the critical condition when a zero DH (*x* = 0) is considered, the exponential function would produce *y* = *a*. The regression coefficient (*a*) ranged from 1.19 to 1.52 according to the experimental data [38]. In other words, when the participant stands quietly on both feet, the force plate would measure 1.19–1.52 BW below each foot according to the exponential function. This is understandable. When DH = 0, the square root function would give PvGRF =* b*. According to the present fit, the intercept *b* values were all in a reasonable interval around the optimal value in most conditions.

Another practical significance of the square root function was its domain (DH ≥ 0). It is impossible for a participant in a traditional landing, rather than jumping movement, to drop from a lower height onto a higher position. It is necessary to reflect this principle in the mathematic formula. Our square root function did it, because a negative DH is senseless to be a radicand. From a mathematical aspect, there was no similar limitation in the exponential function proposed by Yeow et al. [38]. Additionally, the exponential function was based on fitting of measurement on a certain individual. For the same participant, the GRF pattern was very similar when landing from different DHs, but the individual variation among different participants was not considered. Yeow et al. [38] also found great variations in the regression parameters among different participants. In the present study, as many data as possible were gathered together to avoid the influence of individual variations and produce more satisfactory results.

The regression equation for medium frequency was also validated by an experimental measurement done by McNitt-Gray [45]. He measured the resultant GRF on double feet for drop landing, while we modeled the peak value on single foot. Based on the hypothesis proposed by Niu et al. [18] that impact forces on both limbs got their imperfectly simultaneous peaks during landing, the calculated predictive PvGRF on one foot should be slightly higher than half of resultant PvGRF on two feet. As seen in Figure 3, the result was very satisfactory.

### 4.3. Influential Factors

It is well known that women have an increased risk for lower-extremity injury while performing landing movement [15, 28, 51]. Many studies have been done to investigate the biomechanical differences between two genders in landing [6, 8, 29, 32, 41, 42]. As seen in the example listed above, there was no consensus on which one gender has higher PvGRF than the other. This study showed higher PvGRF in men compared to women. Therefore, the higher injury risk in women has no correlation with PvGRF. Scientists should search for a relationship with other parameters, such as joint motion and muscle activities [15, 16]. Based on the finding of higher GRF in men, a former study concluded that men are more likely to transform the kinetic energy to impact [15]. This conclusion was validated, because the same evidence was confirmed in the present study.

A significantly higher mean PvGRF was detected in the platform landing compared to the suspended landing. The platform landing is normally accompanied with a stepping or jumping movement through a reaction force from the platform. This makes the subject leave the platform ahead and upward. In most conditions, the accompanied stepping or jumping movement would produce an extra vertical height, which leads to an actual DH higher than the reported value during platform landing. Relatively, the similar problem is not seen in the study of suspending landing. Therefore, the suspended landing is more reliable than the platform landing when considering the influence of DH.

Wearing shoes significantly decreased PvGRF compared to barefoot. This result is different from that of Shultz et al. [39] and LaPorta et al. [52]. Shultz et al. [39] found significantly higher PvGRF in a shod compared to a barefoot landing, while LaPorta et al. [52] found no significant difference between them. In our opinions, the shoe provides a soft and flat buffer between the foot and the ground and would protect the foot from injury due to a high impact force.

As opposed to the shoe, the ankle stabilizer significantly increased PvGRF during landing. This is consistent with several previous reports [16, 49, 50, 53, 54]. Niu et al. [16] found that semirigid ankle stabilizer could significantly increase PvGRF. Because the ankle stabilizer can effectually protect the ligamentous structure from spraining by controlling the ankle joint, the kinetic energy originally absorbed by joint motion has to be released through increased impact force after stabilizer using [16]. The increased force may influence the biomechanics of bones and cartilages in foot/ankle or even other adjacent regions. It should be seen as an adverse effect of the protection. Therefore, an optimal design should be considered for an ideal prophylactic ankle support to limit the excessive joint motion and meanwhile to allow necessary joint motion.

Surface stiffness had no significant effect on PvGRF. The same conclusion was also obtained in some control studies [3, 55]. Someone may think that there would be a decreased PvGRF with a soft surface because of buffering. In contrast, McNitt-Gray et al. [2] found that using mats significantly increased PvGRF during gymnast landing. The authors considered that participants modulated total body stiffness in responses to changes in landing surface conditions by using a multijoint solution. It is possible that this postural-coordination mechanism and the buffering effect of mats had opposing effects that counteracted the influence on PvGRF.

Hori et al. [56] examined the influences of sample frequency on GRF during CMJ and found that the difference of GRF peaks was minimal between frequencies of 25 Hz and 500 Hz. They thought that sampling could be as low as 200 Hz, depending on the purpose of measurement during CMJ. In the present study, however, PvGRF was significantly smaller when sample frequencies were <1,000 Hz compared with that >1,200 Hz. Therefore, a sample frequency of at least 1,000 Hz was recommended for the application, because no significant difference was found between the medium and high frequency.

### 4.4. Limitations

There are some limitations in the current study. Firstly, the variations among various studies were very large, and it influenced the analyses. To avoid the subjective influence, we tried to use all available data in our analysis. We had to construct three regression equations dependent on sample frequency, because the variation for certain range of sample frequency was greatly less than that for full data. Secondly, only PvGRF values were collected and analyzed in the present study. There are many other parameters to describe the biomechanical feature of landing movement. A complete overview should include all the kinetic, kinematic, and neuromuscular characteristics. An innovative method was constructed in the present study to analyze the influences of various factors on the PvGRF involving all DH situations. This method can be applied to analyze other parameters during landing or other similar questions.

## 5. Conclusion

Twenty-six articles reported PvGRF during a double-leg drop landing from different DHs. Based on the pooled data, a new statistical method was developed to provide the correlation of peak vertical ground reaction force and drop height during two-leg landing and the influences of some factors on the peak vertical ground reaction force regardless of drop height. The PvGRF was not significantly affected by surface stiffness but was significantly greater in men than women, the platform than suspended landing, the barefoot than shod condition, and ankle stabilizer than control condition and higher than lower frequencies.

## Supplementary Material

This document certifies that the paper listed below has been edited to ensure that the language is clear and free of errors. The edit was performed by professional editors at Editage, a division of Cactus Communications.The intent of the author's message was not altered in any way during the editing process. The quality of the edit has been guaranteed, with the assumption that our suggested changes have been accepted and have not been further altered without the knowledge of our editors.

## Figures and Tables

**Table 1:** Characteristics of included articles.

References (authors, year)	Subjects (*n*, sex)	DH (cm)	Landing type	Shoe	Ankle stabilizer	Surface stiffness	Frequency (Hz)
Dufek and Bates, 1990; 1992 [24, 25]	3 M	40/60/100	PF	Shoe	Control	Hard	500
Caster and Bates 1995 [26]	4 M	60	SP	US	Control	Hard	1000
Zhang et al., 2000 [23]	9 M	32/62/103	PF	US	Control	Hard	1000
Riemann et al., 2002 [20]	9 M/5 F	59	PF	Shoe	Both	Hard	1000
Decker et al., 2002 [27]	11 US	60	PF	Shoe	Control	Hard	1200
Decker et al., 2003 [28]	12 M/9 F	60	PF	Shoe	Control	Hard	1200
Seegmiller and McCaw, 2003 [1]	20 F	30/60/90	PF	Barefoot	Control	Soft	960
Kernozek et al., 2005 [29]	15 M/15 F	60	SP	Shoe	Control	Hard	1200
Hodgson et al., 2005 [46]	12 F	61	SP	Shoe	Both	Hard	600
Kulas et al., 2006 [30]	20 F	60	PF	US	Control	Hard	1000
Hoffrén et al., 2007 [22]	5 M/7 F	10/15/20	PF	US	Control	Hard	2000
Zhang et al., 2008 [31]	10 M	30/45/60/75/90	PF	US	Control	Hard	3000
Blackburn and Padua, 2009 [32]	20 M/20 F	60	SP	Shoe	Control	Hard	1000
Gehring et al., 2009 [42]	13 M/13 F	52	PF	Shoe	Control	Hard	1080
Niu et al., 2010 [15]	8 M/8 F	32/52/72	PF	Barefoot	Control	Hard	1000
Sell et al., 2010 [47]	70 M	50	PF	Shoe	Control	Hard	1200
Wallace et al., 2010 [33]	14 M	30/60/90	PF	Shoe	Control	Hard	1200
Niu et al., 2011 [16]	8 M/8 F	32/52/72	PF	Barefoot	Both	Hard	1000
Niu et al., 2011 [18]	8 M/8 F	32/52/72	PF	Barefoot	Control	Hard	1000
Torry et al., 2011 [48]	6 M	40	PF	US	Control	Hard	1200
Chang et al., 2012 [34]	10 M	20/40/60	PF	Barefoot	Control	Hard	1000
Zhang et al., 2012 [49]	5 M/5 F	60	SP	Shoe	Both	Hard	1200
Shultz et al., 2012 [39]	10 M/10 F	45	PF	Barefoot	Control	Hard	1000
Niu and Fan 2013 [3]	8 M/8 F	32/52/72	PF	Barefoot	Control	Both	1000
Simpson et al., 2013 [50]	16 F	43	SP	US	Both	Hard	1200

M: male; F: female; PF: platform landing; SP: suspended landing; US: unspecified in the original article.
